# Network analysis and experimental validation to investigate *chenpi* against functional dyspepsia through TLR4/MyD88 by regulating the gut microbial structure

**DOI:** 10.3389/fphar.2025.1495799

**Published:** 2025-02-13

**Authors:** Jinfang Hu, Xu Wang, Xiaoqiu Guo, Wen Wen, Jin Xue, Zhengzheng Liao, Lihua Chen

**Affiliations:** ^1^ Key Laboratory of Modern Preparation of Traditional Chinese Medicines, Ministry of Education, Jiangxi University of Chinese Medicine, Nanchang, China; ^2^ Department of Pharmacy, the First Affiliated Hospital, Jiangxi Medical College, Nanchang University, Nanchang, Jiangxi, China; ^3^ Center for Experimental Medicine, the First Affiliated Hospital, Jiangxi Medical College, Nanchang University, Nanchang, Jiangxi, China; ^4^ Department of Pharmacy, the Affiliated Hospital of Jiangxi University of Chinese Medicine, Nanchang, Jiangxi, China; ^5^ Jiangxi Center for Drug Certification and Evaluation, Nanchang, Jiangxi, China; ^6^ Formula-pattern Research Center, Jiangxi University of Chinese Medicine, Nanchang, Jiangxi, China; ^7^ State Key Laboratory of Food Science and Technology, China-Canada Joint Laboratory of Food Science and Technology (Nanchang), Key Laboratory of Bioactive Polysaccharides of Jiangxi Province, Nanchang University, Nanchang, Jiangxi, China

**Keywords:** functional dyspepsia, *Citri Reticulatae* Pericarpium, inflammation, TLR4/MyD88, gut microbe

## Abstract

Functional dyspepsia (FD) is a prevalent functional gastrointestinal disorder characterized by the absence of organic lesions; it affects nearly one-fifth of the global population. There is currently no specific drug for treating it. Citri reticulatae Pericarpium (CRP) has been utilized in China for millennia as a therapeutic agent for alleviating bloating and spleen–stomach disharmony. Nonetheless, the curative efficacy and precise molecular mechanisms implicated in FD warrant further investigation. This study aims to address this gap by investigating the potential mechanisms of CRP against FD using HPLC-ESI-QTOF-MS, network analysis prediction, and experimental validation. In this study, 90 CRP metabolites were identified by HPLC-ESI-QTOF-MS; 70 common targets of CRP and FD were extracted, and the top ten overlapped targets included MAPK1, MAPK2, and MAPK3. KEGG enrichment analysis revealed that the MAPK pathways were predominant and involved the TLR4 signaling pathway. *In vivo* experiments demonstrated that after 14 days of treatment, CRP improved body weight, gastric emptying rate, intestinal transit rate, and the pathological structure of the gastric tissue. Serum IL-6, TNF-α, and IL-1β were downregulated, and the expressions of TLR4, MyD88, p-NF-κB, and MAPKs were suppressed in gastric tissue. Furthermore, CRP increased the relative abundance of *Patescibateria* and *Bacteroidota*, accompanied by a reduction in the relative abundance of *Verrucomicrobota* and *Proteobacteria*. In brief, CRP could attenuate dyspepsia by reducing the activation of inflammation-related TLR4/MyD88 and MAPK signaling pathways and by mediating gut microbial structure and composition. This study provides a unique perspective for further research on drugs for treating FD.

## 1 Introduction

Functional dyspepsia (FD), also just termed “dyspepsia”, is a common functional gastrointestinal disease without organic lesion and is prevalent in 21% of the global population (Tack and Talley, 2013; Ford et al., 2015). Epidemiological surveys have shown that two-thirds of FD patients have different clinical manifestations, ranging from postprandial distress syndrome (PDS: postprandial fullness, belching, anorexia, and nausea) to epigastric pain syndrome (EPS: epigastric pain and burning) (Sayuk and Gyawali, 2020). This disease has clinical characteristics of difficult diagnosis, easy recurrence, and high prevalence, and it seriously affects the daily mental and physical health of patients. Although some medications are available for treating FD, many patients do not receive pharmacological treatment due to the nonspecific nature of symptoms. Furthermore, pharmacological interventions for FD may irritate the gastrointestinal mucosa, leading to adverse reactions during treatment (Talley, 2016). Therefore, developing a new type of clinically safe and effective drug for treating FD has always been a popular research topic for scientists.

The exact pathogenesis of FD is still unclear, but gender, smoking, the use of non-steroidal anti-inflammatory drugs, *Helicobacter pylori* infection, the gut–brain axis, mucosal and immune function, dietary and lifestyle factors, central nervous system (CNS) processing, genetic predisposition, and hormonal dysregulation are probably related to its progression (Ford et al., 2020). In addition, much research indicates a close relationship between psychological factors and the onset and progression of FD (Wolf, 1943; Jiang and Travagli, 2020). Existing studies demonstrate that the incidence of depression, tension, anxiety, insomnia, and emotional disorders in FD patients are up to 42%–61% (Wauters et al., 2020). Another study also found a positive correlation between the severity of FD symptoms and negative emotions (Mak et al., 2020). Depression and anxiety can lead to decreased vagal nerve activity, delayed gastric emptying, and high sensitivity of the gastrointestinal tract, resulting in recurrent FD symptoms. Notably, the imbalance of intestinal flora and its metabolites caused by psychological stress plays an unprecedented large role in the morbidity and pathological process of gastrointestinal diseases (Bian et al., 2021). Disordered gut microbiota homeostasis could alter the intestinal barrier function, promoting the penetration of harmful products through the intestinal mucosal barrier and thereby affecting gastric function (Wang et al., 2020). Apart from the above role, disrupted intestinal microbiota homeostasis could activate macrophages, which are ubiquitous in the gastrointestinal tract, through its metabolites to release pro-inflammatory cytokines, further exacerbating gastrointestinal dysfunction (Jing et al., 2021). Previous research has found that the degree of infiltration in the gastric mucosa of FD patients was significantly higher than that of normal individuals (Arisawa et al., 2007; Vanheel et al., 2017). Furthermore, the elevated levels of TNF-a, IL-1β, and IL-6 in the serum of FD patients can stimulate mast cells in the gastric mucosa, causing impaired gastrointestinal motility and visceral sensation (Gwee, 2010). (Ciesielska et al., 2021) revealed that the inflammatory response depends on the activation of the toll-like receptor 4 (TLR4)/myeloid differentiation factor 88 (MyD88) pathway in gastrointestinal diseases. The activation of the TLR4/MyD88 pathway in turn promotes the release of inflammatory factors that affect the secretion of brain–gut axis peptides closely related to gastric motility (Ford et al., 2020). Therefore, decreasing TLR4/MyD88-mediated inflammatory responses by repairing intestinal homeostasis and its metabolites may be an effective way of treating FD in clinical practice.

Citri reticulatae Pericarpium (CRP), commonly called by the Chinese name *chenpi*, is derived from the mature fruit peel of *Citrus reticulata* Blanco. CRP has been widely used as a food seasoning and in traditional Chinese medicine (TCM) for 3,000 years (Zheng et al., 2020; Zou et al., 2022). Commonly used for promoting *qi* circulation, CRP can alleviate negative emotions such as anxiety, depression, and irritability. According to TCM theory, CRP has *qi*-regulating effects and is used to treat diseases related to “stagnation” characteristics of *qi*, such as food stagnation and distension (Yu et al., 2018). CRP has also been noted for its function in treating food bloating and spleen–stomach disharmony (deficiency) since its first inclusion in the Chinese Pharmacopoeia (1953 edition). In recent years, CRP extracts have been identified as rich in flavonoids, alkaloids, and volatile oils and verified in a variety of biological functions, including anti-inflammatory (Ho and Kuo, 2014), anti-fibrosis (Perez-Vargas et al., 2014), and anti-allergic (Hagenlocher et al., 2017). However, there are few studies on modern pharmacological research on the treatment of gastrointestinal dysfunction induced by emotional depression with CRP. Therefore, we here designed an experiment to explore the therapeutic effects and mechanisms of CRP on FD caused by hypoemotivity in a rat model.

## 2 Materials and methods

### 2.1 Materials

Dried CRP (batch number: R2301001) was obtained from Jianchangbang Pharmaceutical Co., Ltd. (Nanchang city, China). ELISA kits for IL-6, TNF-α and TNF-α were purchased from Biosciences (Pharmingen, BD Biosciences, United States). The primary antibodies for TLR4, MyD88, NF-κB, p-NF-κB, and β-actin used in Western blot analysis were purchased from Cell Signaling Technology (CST, United States). HRP-conjugated anti-rabbit antibodies were acquired from Santa Cruz Biotechnology, United States. Formic acid, methanol, and acetonitrile were acquired from Merck Co. (New Jersey, United States). Distilled water was provided by Hangzhou Wahaha Co., Ltd., China. Other reagents used were analytical grade.

### 2.2 Preparation of CRP extract

We placed 1 kg of dried CRP in ten-times distilled water for 2 h, reflux extracted three times, each time for 1 h, and filtered. The CRP extracts were combined, concentrated under negative pressure conditions, freeze-dried, and then stored at −20°C.

### 2.3 Composition analysis of CRP extract

The composition analysis of CRP was extracted by HPLC-ESI-QTOF-MS. The chromatographic system was Shimadzu Nexera X2, and the column was Shim-pack GIST C18 column (2.1 mm × 75 mm, 2 µm). The flow rate was set at 0.3 mL/min; The column temperature was 35°C; the injection volume was 2 μL, and the gradient elution of 0.1% formic acid distilled water and 0.1% formic acid in acetonitrile (B) was set to the mobile phase (Table 1).

**TABLE 1:** Gradient elution ratio of mobile phase.

Time (min)	A (0.1% formic acid water)	B (0.1% formic acid acetonitrile)
0	98	2
2.0	95	5
3.0	87	13
8.0	78	22
9.0	65	35
13.0	65	35
21.0	55	45
22.0	5	95
30.0	5	95
30.1	98	2
38.0	98	2

An AB Scicx Triple TOF 5600+ equipped with an electrospray ionization source (ESI) was used for MS/MS^2^ data acquisition in positive and negative ionization modes. The conditions are shown in Table 2. The range of primary mass spectrometry acquisition was m/z 100–1,500 Da.

**TABLE 2:** The optimized MS parameters.

Parameters	Positive mode	Negative mode
ISVF	5500 V	−4500 V
TEM	550°C	550°C
DP	100 V	−80 V
CE	30 eV	−10 eV
Gas 1	50 psi	50 psi
Gas 2	50 psi	50 psi
Curtain gas	30 psi	30 psi

Ion spray voltage floating (ISVF), turbo spray temperature (TEM), declustering potential (DP), collision gas (CE), nebulizer gas (Gas 1), heater gas (Gas 2), and curtain gas for positive and negative ionization mode.

### 2.4 Network analysis

The targets of identified metabolite were obtained from the Encyclopedia of Traditional Chinese Medicine (ETCM) database and SwissTargetPrediction database. The disease targets of FD were obtained from the Online Mendelian Inheritance in Man (OMIM) database and GeneCards database. The “metabolite-target” network was constructed by using Cytoscape 3.7.1 software based on the intersecting targets between disease targets and the active metabolites targets. The potential targets were analyzed using String’s Protein–Protein Interaction (PPI) database (https://cn.string-db.org/) and Cytoscape 3.7.1 software. Finally, gene ontology (GO) and Kyoto Encyclopedia of Genes and Genomes (KEGG) enrichment analyses of intersecting genes in the previous stage were performed by the clusterProfiler package for R.

### 2.5 Animal experiments and treatment

We purchased 42 specific-pathogen-free (SPF) SD male rats (180–200 g) from GemPharmatech Co., Ltd. (NO: SCXK (su) 2023-0009, Nanjing, Jiangsu, China). All rats were placed in a room with controllable temperature and humidity in 12-h light/dark cycle and acclimated for 7 days before experiment. All animal experiments were performed according to the animal ethics guidelines of the Animal Experimentation of the First Affiliated Hospital, Jiangxi Medical College, Nanchang University (reference number: CDYFY-IACUC-202306QR023). The model of FD was built by semi-starvation followed by tail damping, binding, forced exercise fatigue, and provocation four times (30 min each time) a day for 14 days (Liang et al., 2018). After the model was established, the FD rats were randomly divided into five groups with an oral supplement of 0.5% distilled water (model group), 0.1 g/kg of CRP extract (CRP -L group), 0.2 g/kg of CRP extract (CRP -M group), 0.4 g/kg of CRP extract (CRP-H group), and 6 mg/kg of domperidone (DOM group) for 14 days. The orally supplement volume was converted to 0.1 mL/10 g according to body weight. During the entire experiment, the rats were free to drink water and were fed a normal chow diet.

### 2.6 Collection and pre-treatment of rat samples

Feces from all the experiment rats were collected after the last day of administration and stored at −80°C until analysis. After the second day of fasting for 12 h, blood was gathered from the femoral arteries after euthanasia and placed in 1.5-mL non-anticoagulation test tubes. The whole blood was centrifuged at 3,500 rpm for 10 min at 25°C, and the supernatant was obtained and stored at −80°C. Next, the abdominal cavity was opened to expose the entire stomach, and then the pyloric orifice and gastric cardia were ligated. The entire gastric tissue was weighed using a precision electronic analytical balance (JA1003, Pruiste, China). Then, the content of stomach was cleaned out by 0.9% saline solution and weighed after drying. The gastric remnant rate and the propulsive intestinal rate were calculated as per Zhu et al. (2020). The gastric tissue was fixed in 4% paraformaldehyde for observing pathological changes or stored −80°C for biochemical testing.

### 2.7 Pathological examination assay

The gastric tissue was prepared into paraffin sections after dehydration and embedding. The slices were dewaxed, stained with hematoxylin (5 min) and eosin (5 min), and observed under a Nikon Eclipse E100 microscope.

### 2.8 Enzyme-linked immunosorbent assay

The IL-6, TNF-α, and IL-1β sera were detected by ELISA kits according to the manufacturer’s instructions. The sample optical density (OD) values were obtained by a microplate reader, and the concentrations of IL-6, TNF-α, and IL-1β sera were analyzed by their respective standard curves.

### 2.9 RNA purification and real-time qPCR

Total RNA was extracted using 500 μL TRIzol Reagent in a 1.5-mL EP tube without RNAase, followed by reverse transcription with 2 μL 10×RT buffer, 0.8 μL 25×dNTP Mix, 2 μL 10×RT Random Primers, 1 μL MultiScribe reverse transcriptase, 1 μL RNAase Inhibitor, and 3.2 μL nuclease-free H_2_O. Quantitative analysis for mRNA expression was performed using the 2^−ΔΔCt^ method, and the mRNA expression of GADPH was chosen as the internal reference. The sequences of the primers for PCR used are shown in Table 3.

**TABLE 3:** Primer sequence list for RT-qPCR.

Gene	Forward primer	Reverse primer
IL-1β	CTA​TGG​CAA​CTG​TCC​CTG​AAC	GCT​TGG​AAG​CAA​TCC​TTA​ATC​T
IL-6	CAC​TTC​ACA​AGT​CGG​AGG​CT	TCT​GAC​AGT​GCA​TCA​TCG​CT
TNF-α	GCC​ACC​ACG​CTC​TTC​TGT​C	GCT​ACG​GGC​TTG​TCA​CTC​G

### 2.10 Western blotting analysis

The gastric tissue protein was extracted and the concentration quantified using a bicinchoninic acid (BCA) kit. The protein samples were separated by 10%–12% sodium salt polyacrylamide gel electrophoresis (SDS-PAGE) and then transferred to polyvinylidene fluoride (PVDF) membranes. The membranes containing proteins were blocked 2.5 h by 7.5% skim milk, and then incubated with primary antibodies at 4°C. The next day, the membranes were incubated with secondary antibodies for 2 h at 25°C. Finally, the protein bands were observed by gel imaging system (Bio-Rad, California, United States) and quantified using ImageJ.

### 2.11 16S rRNA sequencing assay

16S rRNA sequencing was performed by Majorbio Co., Ltd., (Shanghai, China). Total DNA from the feces was extracted, specific primers with Barcode were synthesized, PCR amplification was conducted, it was then purified and quantified, and a sequencing library built. Finally, the library was sequenced by PacBio Sequel. The raw data obtained were pre-processed and bioinformatics analysis performed.

### 2.12 Statistical analysis

GraphPad Prism 9.0 version was employed for statistical analyses. The differences between the groups were detected by one-way analysis of variance (ANOVA). Differences of *p* < 0.05 were considered statistically significant, and the data were presented by the mean and the standard error of means (SEM).

## 3 Result

### 3.1 Target prediction in network analysis of CRP metabolites for treating FD

#### 3.1.1 CRPmetabolites

**FIGURE 1:**
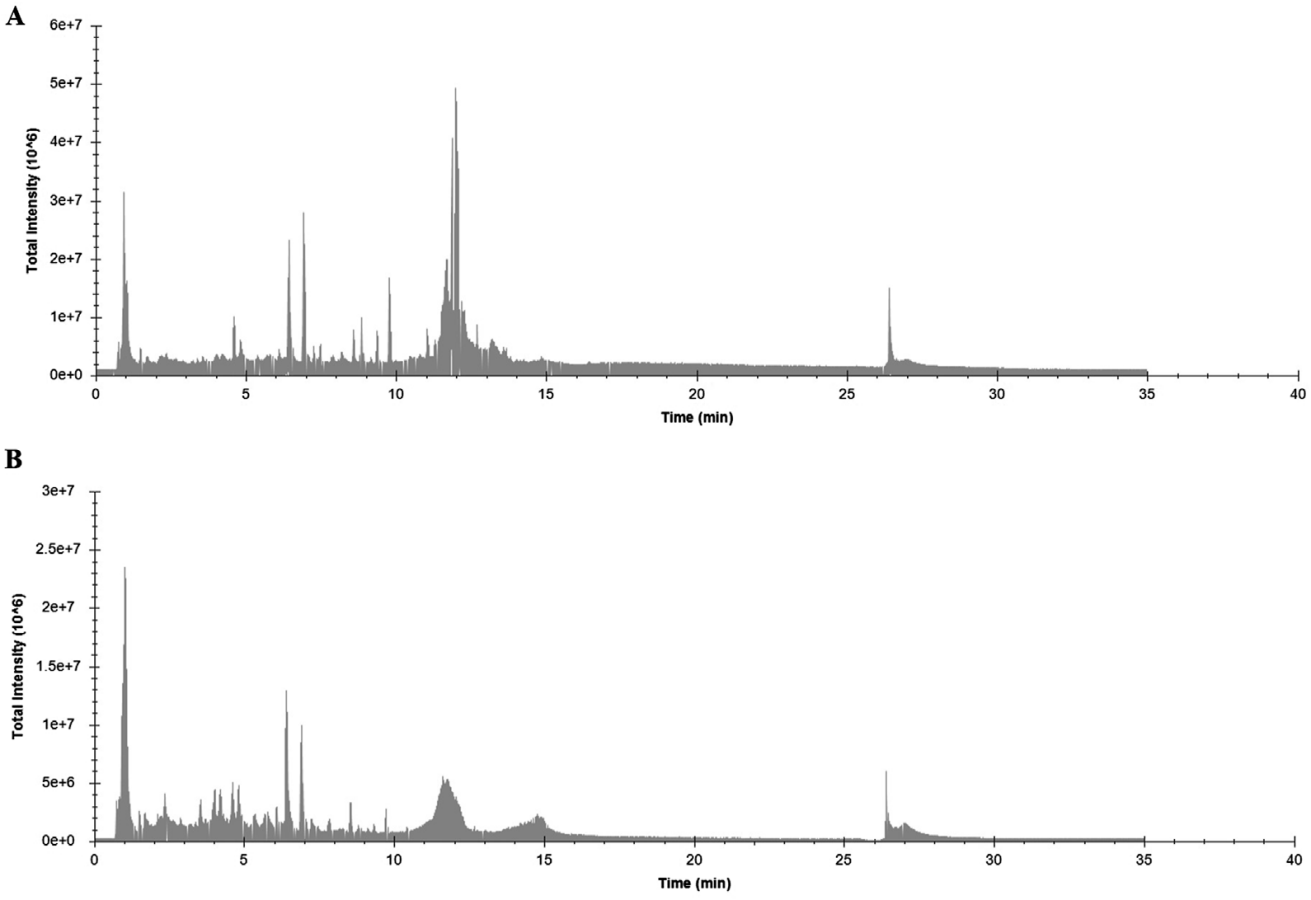
Base peak intensity (TIC) chromatograms in positive **(A)** and negative **(B)** ion modes of CRP extract.

The HPLC-ESI-QTOF-MS method was employed to investigate CRP metabolites. CRP ion chromatograms in negative and positive ion modes are shown in Figure 1. We identified 90 metabolites, including 45 flavonoids, 25 alkaloids, two carbohydrates, one organic acid, two phenylpropanoids, five phenols, two steroids, seven fatty acids, one sesquiterpene, and one other. The identified chemicals are listed in Table 4.

**TABLE 4:** Preliminary identification of compounds in CRP extract (negative/positive).

No.	RT (min)	Molecular weight (Da)	Formula	Ionization mode	Identify
1	0.87	175.1189	C_6_H_14_N_4_O_2_	[M+H]^+^	Arginine
2	0.92	333.0668	C_28_H_34_O_15_	[M+H]^+^	Hesperidin
3	0.96	179.0555	C_6_H_12_O_6_	[M+CH_3_COO]^−^	Sorbose
4	1.01	116.0698	C_5_H_9_NO_2_	[M+H]^+^	L-proline acid
5	1.05	144.1020	C_7_H_13_NO_2_	[M+H]^+^	(2R)-6-methylpiperidine-2-carboxylic acid
6	1.05	130.0858	C_6_H_11_NO_2_	[M+H]^+^	Pipecolic acid
7	1.15	365.1056	C_19_H_18_O_6_	[M+Na]^+^	Methylophiopogonanone A
8	1.45	103.0033	C_3_H_4_O_4_	[M−H]^−^	Malonic acid
9	1.51	244.0909	C_9_H_13_N_3_O_5_	[M+H]^+^	Cytidine
10	1.97	245.0773	C_9_H_12_N_2_O_6_	[M+H]^+^	Uridine
11	1.99	113.0342	C_4_H_4_N_2_O_2_	[M+H]^+^	Uracil
12	2.20	266.0881	C_10_H_13_N_5_O_4_	[M−H]^−^	Adenosine
13	2.20	346.0544	C_10_H_12_O_7_N_5_P	[M+H]^+^	Guanosine cyclic monophosphate
14	2.27	282.0837	C_10_H_13_N_5_O_5_	[M−H]^−^	Guanosine
15	2.68	166.0857	C_9_H_11_NO_2_	[M+H]^+^	Phenylalanine
16	3.14	389.1758	C_22_H_26_N_2_O_3_	[M+K]^+^	Hirsuteine
17	3.38	203.0832	C_11_H_12_N_2_O_2_	[M−H]^−^	Tryptophan
18	3.39	146.0593	C_9_H_7_NO	[M+H]^+^	Indole-3-carboxaldehyde
19	3.57	757.2163	C_33_H_41_O_20_	[M+H]^+^	Cyanidin 3-glucosylrutinoside
20	3.64	471.1259	C_22_H_24_O_10_	[M+K]^+^	Isosakuranin
21	3.70	490.2654	C_25_H_41_NO_7_	[M+Na]^+^	Delsoline
22	3.73	204.1228	C_9_H_17_NO_4_	[M+H]^+^	Acetyl-L-Carnitine
23	4.00	465.0999	C_21_H_20_O_12_	[M+H]^+^	Isoquercitrin
24	4.08	355.1040	C_16_H_18_O_9_	[M+H]^+^	Cryptochlorogenic acid
25	4.19	609.1454	C_27_H_30_O_16_	[M−H]^−^	Kaempferol 3-O-sophoroside buten-1-ylidene)cyclohexyl β-Dglucopyranoside
26	4.58	809.2098	C_34_H_42_O_21_	[M+Na]^+^	Isorhamnetin 3-sophoroside-7-rhamnoside
27	4.80	623.1631	C_34_H_42_O_21_	[M−H]^−^	Isorhamnetin-3-O-galactoside-6″-rhamnoside
28	4.83	765.2229	C_27_H_30_O_16_	[M+Na]^+^	Troxerutin
29	4.83	273.0733	C_27_H_32_O_14_	[M+H]^+^	Naringenin
30	5.50	685.2664	C_32_H_50_O_18_	[M−H]^−^	Secoisolariciresinol diglucoside
31	5.65	587.1414	C_27_H_30_O_15_	[M+Na]^+^	Isoshaftoside
32	5.67	433.1130	C_21_H_20_O_10_	[M+H]^+^	Sophoricoside 3,4,5-trihydroxy-6-(hydroxymethyl)oxan-2-yl]oxybutanoic acid
33	5.72	163.0390	C_9_H_8_O_3_	[M−H]^−^	p-coumaric acid eicosatetraenoic acid
34	5.80	303.0513	C_15_H_10_O_7_	[M+H]^+^	Quercetin
35	5.80	611.1626	C_27_H_30_O_16_	[M+H]^+^	Rutine
36	5.89	573.1578	C_26_H_30_O_13_	[M+Na]^+^	Isoliquiritin apioside
37	5.90	431.0955	C_21_H_20_O_10_	[M−H]^−^	3-genistein-8-O-glucoside
38	5.94	739.2057	C_33_H_40_O_19_	[M−H]^+^	Kaempferol-3-O-galactoside-6″-rhamnoside-3‴-rha
39	5.97	763.2023	C_33_H_40_O_19_	[M+Na]^+^	Apigenin-7-O-(2G-O-rhamnosyl)gentiobioside
40	6.07	463.0857	C_21_H_20_O_12_	[M−H]^−^	Isoquercetin
41	6.08	487.0853	C_21_H_20_O_12_	[M+Na]+	Myricitrin
42	6.09	755.1807		[M−H]^−^	Quercetin 3-O-[2″-O-(6‴-O-p-coumaroyl)-β-D-glucopyranosyl]-a-L-rhamnopyranoside
43	6.09	303.0467	C_15_H_11_O_7_	[M+Na]^+^	Delphinidin
44	6.18	193.0512	C_10_H_10_O_4_	[M−H]^−^	Ferulic acid
45	6.29	461.1075	C_22_H_22_O_11_	[M−H]^−^	Scoparin
46	6.35	807.2001	C_28_H_32_O_15_	[M+Na]^+^	Spinosin B
47	6.40	287.0582	C_15_H_10_O_6_	[M+H]^+^	Luteolin
48	6.40	449.1133	C_21_H_20_O_11_	[M+H]^+^	Kaempferol-4′-glucoside
48	6.41	579.1725	C_21_H_22_O_10_	[M−H]^−^	Naringenin-7-O-rutinoside
49	6.41	615.1470	C_27_H_32_O_14_	[M+Cl]^−^	Naringin
50	6.44	295.1274	C_14_H_31_NO	[M+H]^+^	N, N-dimethyldodecylamine N-oxide
51	6.52	577.1564	C_27_H_30_O_14_	[M−H]^−^	Rhoifolin
52	6.72	607.1665	C_28_H_32_O_15_	[M−H]^−^	Diosmin
53	6.90	479.1197	C_22_H_22_O_12_	[M+H]^+^	Isorhamnetin-3-O-β-D-glucoside
54	6.92	301.0703	C_16_H_14_O_6_	[M−H]^−^	Hesperetin
55	7.00	511.3435	C_30_H_48_O_5_	[M+Na]^+^	Asiatic acid 1,2,4a-trimethyl-1,2,3,4,4a,7,8,8aoctahydro-1-naphthalenyl]-3-methylpentanoic acid
56	7.41	186.2212	C_12_H_27_N	[M+H]^+^	N, N-dimethyldecylamine bis[3,4,5-trihydroxy-6-(hydroxymethyl)tetrahydro-2H-pyran-2-yl]-4H-chromen-4-one
57	8.45	257.0819	C_15_H_12_O_4_	[M+H]^+^	Pinocembrin
58	8.56	639.1912	C_28_H_34_O_14_	[M+HCOO]^−^	Isosakuranetin-7-O-rutinoside
59	8.56	285.0771	C_16_H_14_O_5_	[M−H]^−^	Isosakuranetin
60	8.56	593.1866	C_28_H_34_O_14_	[M−H]^−^	Poncirin
61	8.59	287.0918	C_16_H_14_O_5_	[M+H]^+^	Sakuranetin
62	9.77	747.2095	C_33_H_40_O_18_	[M+Na]^+^	3-feruloyl-1-sinapoyl sucrose
63	11.05	395.1107	C_20_H_20_O_7_	[M+Na]^+^	Tangeritin
64	11.31	274.2741	C_16_H_35_NO_2_	[M+H]^+^	Lauryldiethanolamine
65	11.36	230.2478	C_16_H_18_O_9_	[M+H]^+^	Chlorogenic acid
66	11.49	425.1224	C_21_H_22_O_8_	[M+Na]^+^	Nobiletin
67	11.52	200.2381	C_13_H_29_N	[M+H]^+^	N-methyldodecylamine
68	11.59	214.2535	C_14_H_31_N	[M+H]^+^	N, N-dimethyldodecylamine
69	11.64	365.0998	C_19_H_18_O_6_	[M+Na]^+^	6-demethoxytangeretin
70	11.68	258.2791	C_16_H_35_NO	[M+H]^+^	N, N-dimethyltetradecylamine-N-oxide
71	12.00	455.1309	C_22_H_24_O_9_	[M+Na]^+^	3, 3′, 4′, 5, 6, 7, 8-heptamethoxyflavone
72	12.06	395.1080	C_20_H_20_O_7_	[M+Na]^+^	Isosinensetin
73	12.10	429.1132	C_20_H_22_O_9_	[M+Na]^+^	Oxyresveratrol 3′-O-β-D-glucopyranoside
74	12.24	411.1028	C_20_H_20_O_8_	[M+Na]^+^	3′-demethylnobiletin
75	12.25	337.0980	C_18_H_18_O_5_	[M+Na]^+^	Flavokawain A
76	12.34	417.3496	C_27_H_44_O_3_	[M+H]^+^	Tigogenin
77	12.43	295.2256	C_18_H_32_O_3_	[M−H]^−^	9-HODE
78	12.47	599.1137	C_30_H_24_O_12_	[M+Na]^+^	Procyanidin A2
79	12.64	383.2015	C_22_H_26_N_2_O_4_	[M+Na]^+^	Corynoxeine
80	12.67	205.1599	C_14_H_22_O	[M−H]^+^	4-octylphenol
81	12.79	299.2550	C_18_H_36_O_3_	[M−H]^−^	12-hydroxyoctadecanoic acid
82	13.02	219.1731	C_15_H_22_O	[M+H]^+^	α-cyperone
83	13.18	277.2169	C_18_H_30_O_2_	[M−H]^+^	γ-linolenic acid
84	13.67	279.2319	C_18_H_32_O_2_	[M−H]^+^	Linoleate
85	14.22	255.2343	C_16_H_32_O_2_	[M−H]^−^	Hexadecanoic acid
86	14.35	281.2498	C_18_H_34_O_2_	[M−H]^−^	Oleic acid
87	15.05	284.2944	C_18_H_37_NO	[M+H]^+^	Stearamide
88	15.46	283.2627	C_18_H_36_O_2_	[M−H]^−^	Stearic acid
89	15.74	265.1505	C_12_H_26_O_4_S	[M−H]^−^	Lauryl sulfate
90	26.34	104.1067	C_4_H_9_NO_2_	[M+H]^+^	2-aminobutyric acid

#### 3.1.2 Common targets of CRP and FD

**FIGURE 2:**
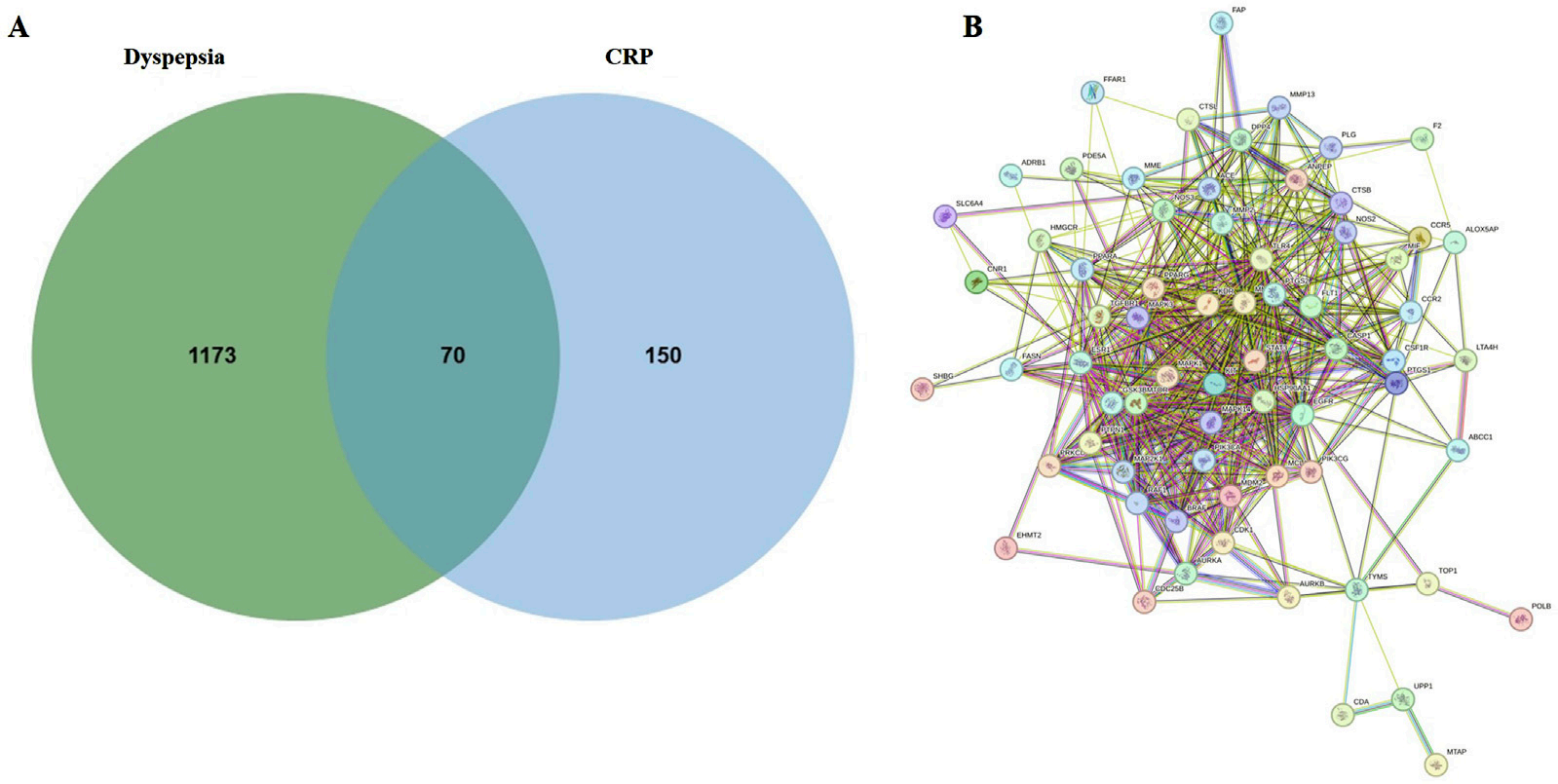
Target of CRP and FD. **(A)** Venn diagram of potential targets in CRP and FD. **(B)** PPI network of the putative targets.

Based on the results of composition analysis, 220 targets of CRP in the ETCM system were found after being corrected by the UniProt database. We identified 1,243 targets of FD from the PubMed and Genecard databases after dropping duplicate records. Figure 2A uses a Venn diagram to show 70 common targets of CRP and FD which were discovered. Subsequently, a PPI network was built based on online String databases. According to the B degree value, the top-ten targets of CRP and FD were MAPK1, HSP90AA1, MMP9, mTOR, ESR1, MAP2K1, PTGS2, EGFR, STAT3, and MAPK3 (Figure 2B). More importantly, these targets are closely related to inflammatory response, especially MAPK1, MAP2K1, and MAPK3.

#### 3.1.3 GO and KEGG analyses of the common the targets

**FIGURE 3:**
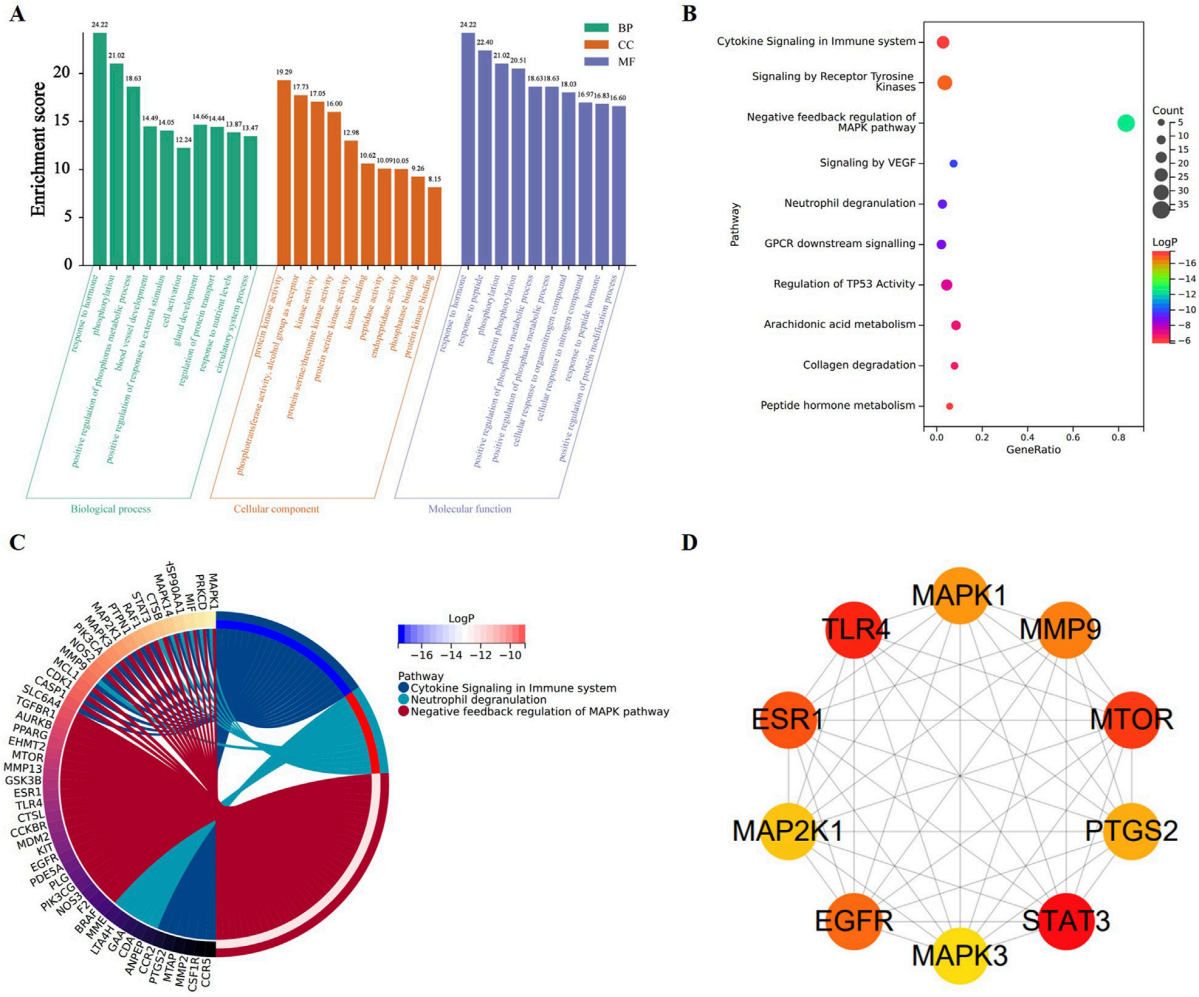
Prediction of potential targets and construction of CRP against FD PPI networks. **(A)** Top 10 remarkably enriched terms on biological process, cellular component, and molecular function from GO analysis. **(B)** Top 10 remarkably enriched pathways from KEGG analysis. **(C)** Chord diagram of potential target. **(D)** PPI network of core targets, featuring 10 nodes (for interpretation of the references to color in this figure legend, the reader is referred to the Web version of the article).

To better understand the role of the common targets, GO and KEGG enrichment analyses were performed. The GO results were obtained via biological process (BP), cellular component (CC), and molecular function (MF), which exhibited top 10 was included (Figure 3A). The KEGG results showed that the MAPK pathways were the principal pathways, but also involved the TLR4 signaling pathway and a variety of inflammatory-related diseases (Figure 3B). We also established a chord diagram to demonstrate target enrichment in cytokine signaling in the immune system, neutrophil degranulation, and negative feedback regulation of the MAPK pathway (Figure 3C). The core targets were comprehensively considered through PPI network, GO, and KEGG analyses, allowing us to select MAPK1, MAP2K1, MAPK3, and TLR4 as pivotal targets within CRP for addressing FD (Figure 3D).

### 3.2 CRP improved the body weight, gastric emptying rate, propulsive intestinal rate, and pathological structure of the gastric tissue of FD rats

**FIGURE 4:**
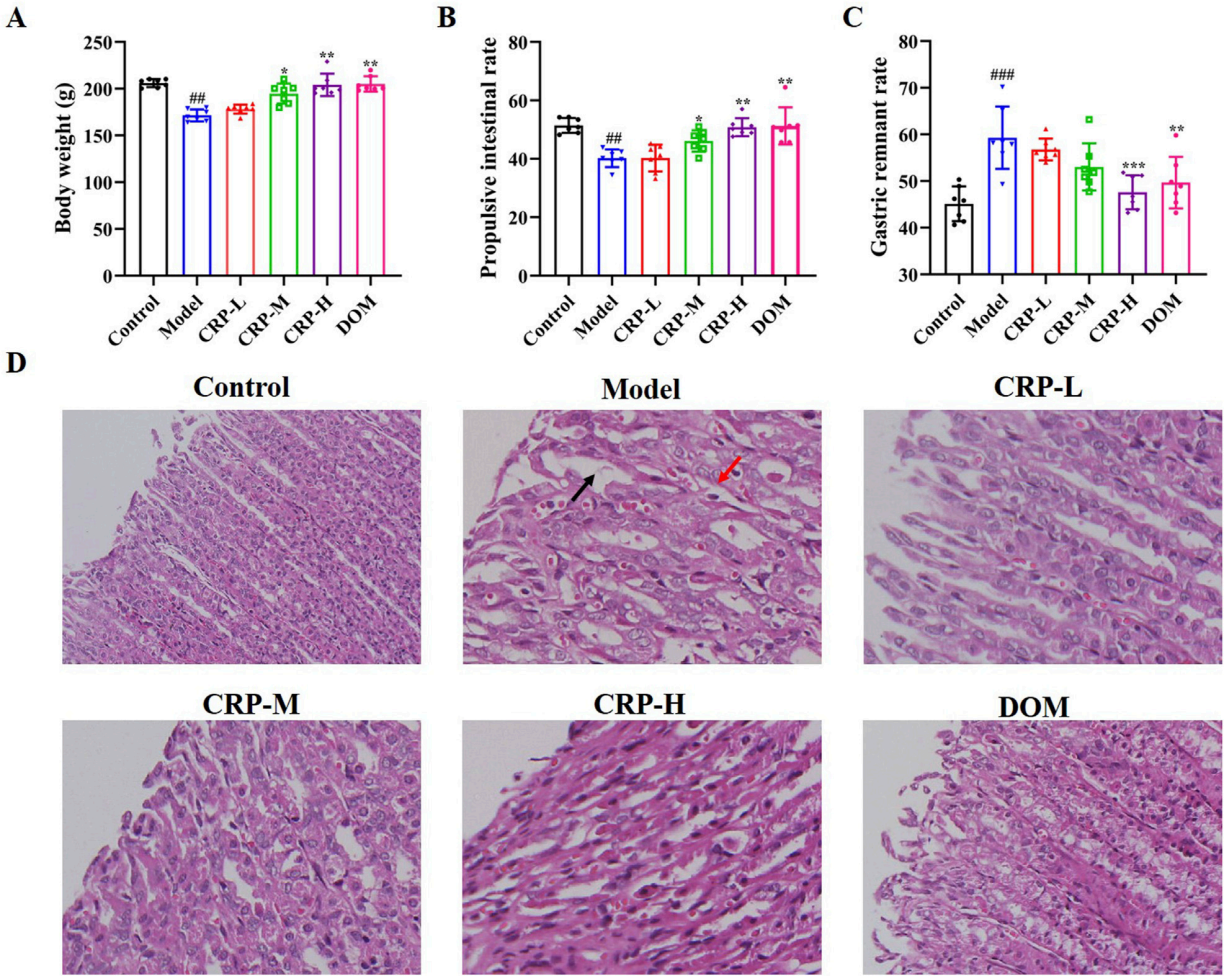
Effects of CRP on gastrointestinal motility disorders in FD rats. Body weight **(A)**, propulsive intestinal rate **(B)** and gastric remnant rate **(C)** in rats. H&E staining of gastric antrum tissue. Scale bar: 100 μm. Magnification ×20 **(D)**. Values expressed as mean ± SEM. ^##^ *P* < 0.01, ^###^ *P* < 0.001 vs. control group; ^*^ *P* < 0.05, ^**^ *P* < 0.01 vs. model group.

As shown in Figure 4, a significant reduction was observed in the body weight and propulsive intestinal rate of rats in the FD group (*P* < 0.01), and a significant increase was observed in the gastric remnant rate (*P* < 0.001) compared to the blank group. After 2 weeks of CRP administration, the FD rats exhibited a significant increase in body weight and propulsive intestinal rate, along with a decrease in gastric remnant rate, at doses of 0.2 and 0.4 g/kg. (Figures 4A–C). H&E staining of the gastric tissue in the control rats revealed intact gastric mucosal layer cells, with clear contours and no obvious inflammatory infiltration (Figure 4D). However, in the FD rats, the gastric tissue structure had slight abnormal scattering, and some of the epithelial cells in the mucosal layer were shed (black arrow). In addition, the submucosal layer was swollen, accompanied by slight inflammatory cell infiltration (red arrow). In contrast, the CRP and DOM groups exhibited a mostly normal gastric tissue structure, with tightly and neatly arranged mucosal layer cells. Additionally, inflammatory infiltration in the epithelial cells was reduced, suggesting that CRP reversed the pathological changes in FD rat gastric tissue. The data strongly indicate that CRP could improve the body weight, gastric remnant rate, propulsive intestinal rate, and gastric pathological structure of FD rats.

### 3.3 CRP eliminated inflammatory cytokines in FD rats

**FIGURE 5:**
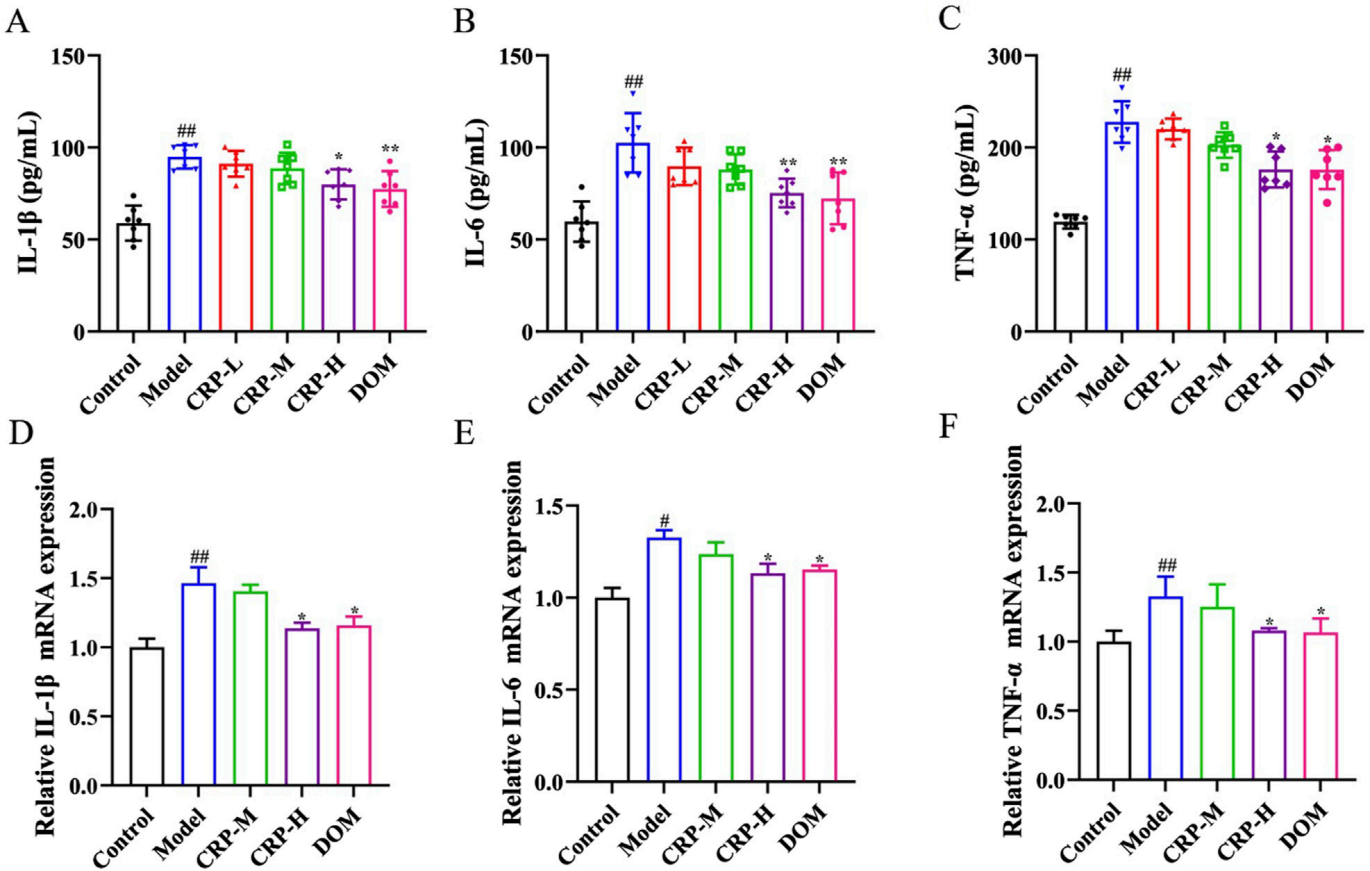
CRP inhibited the inflammation response in FD rats. **(A–C)** Content of IL-1β, IL-6, and TNF-α in serum (n = 7). **(D–F)** mRNA expression of IL-1β, IL-6, and TNF-α in gastric antrum tissue (n = 3). Values expressed as mean ± SEM. ^#^ *P* < 0.05, ^##^ *P* < 0.01 vs. control group; ^*^ *P* < 0.05, ^**^ *P* < 0.01 vs. model group.

In order to further understand the therapeutic effect of CRP on FD rats, we used ELISA kits and the RT-qPCR method to analyze the levels of inflammatory cytokine factors in serum and gastric tissue. The contents of IL-1β, IL-6, and TNF-α in serum were detected by ELISA kits, and the result demonstrated that CRP-H downregulated the level of inflammatory cytokines (*P* < 0.05, *P* < 0.01 and *P* < 0.05—Figures 5A–C). In addition, the inflammatory cytokines were measured in gastric tissue by RT-qPCR, and the data demonstrated that CRP-H could reduce the mRNA expression of IL-1β, IL-6, and TNF-α (*P* < 0.05—Figures 5D–F).

### 3.4 CRP inhibited TLR4/MyD88- and MAPKs-dependent signaling pathway

**FIGURE 6:**
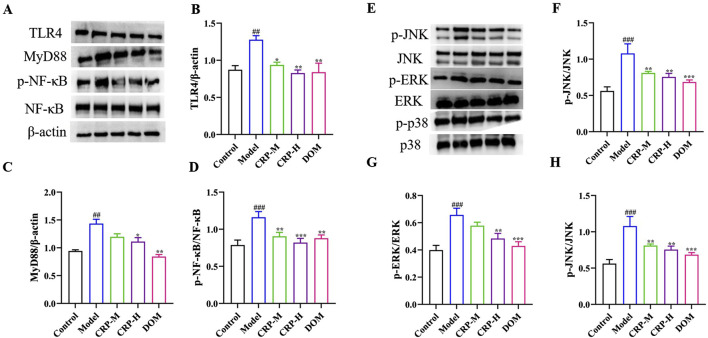
Effects of CRP on the expressions of TLR4/MyD88- and MAPK-related proteins. Representative Western blot **(A, E)** and quantitative analysis **(B–D, F–H)** of gastric antrum shown. Values expressed as mean ± SEM (n = 3). ^##^ *P* < 0.01, ^###^ *P* < 0.001 vs. control group; ^*^ *P* < 0.05, ^**^ *P* < 0.01, ^***^ *P* < 0.001 vs. model group.

FD often accompanies the occurrence of gastric tissue inflammation (Zhang et al., 2022). The TLR4/MyD88 pathway is an important effector of inflammatory response signal transduction via NF-κB and MAPKs. Therefore, we used Western blotting to detect the TLR4/MyD88 pathway and its dependent signals. As shown in Figure 6, the results indicated that TLR4 and MyD88 expression in the model group were higher than those in normal rats (*P* < 0.01). Compared with the control rats, the contents of p-NF-κB/NF-κB in the model rats significantly increased (*P* < 0.001). However, CRP at medium and high doses could reverse this change. In addition, CRP in MAPK family proteins decreased the upregulation of p-JNK/JNK, p-ERK/ERK, and p-P38/P38 levels in FD rats (*P* < 0.001). Therefore, we may link the therapeutic effect of CRP on FD with its anti-inflammatory effect.

### 3.5 CRP improves the diversity of gut microbiota in FD rats

**FIGURE 7:**
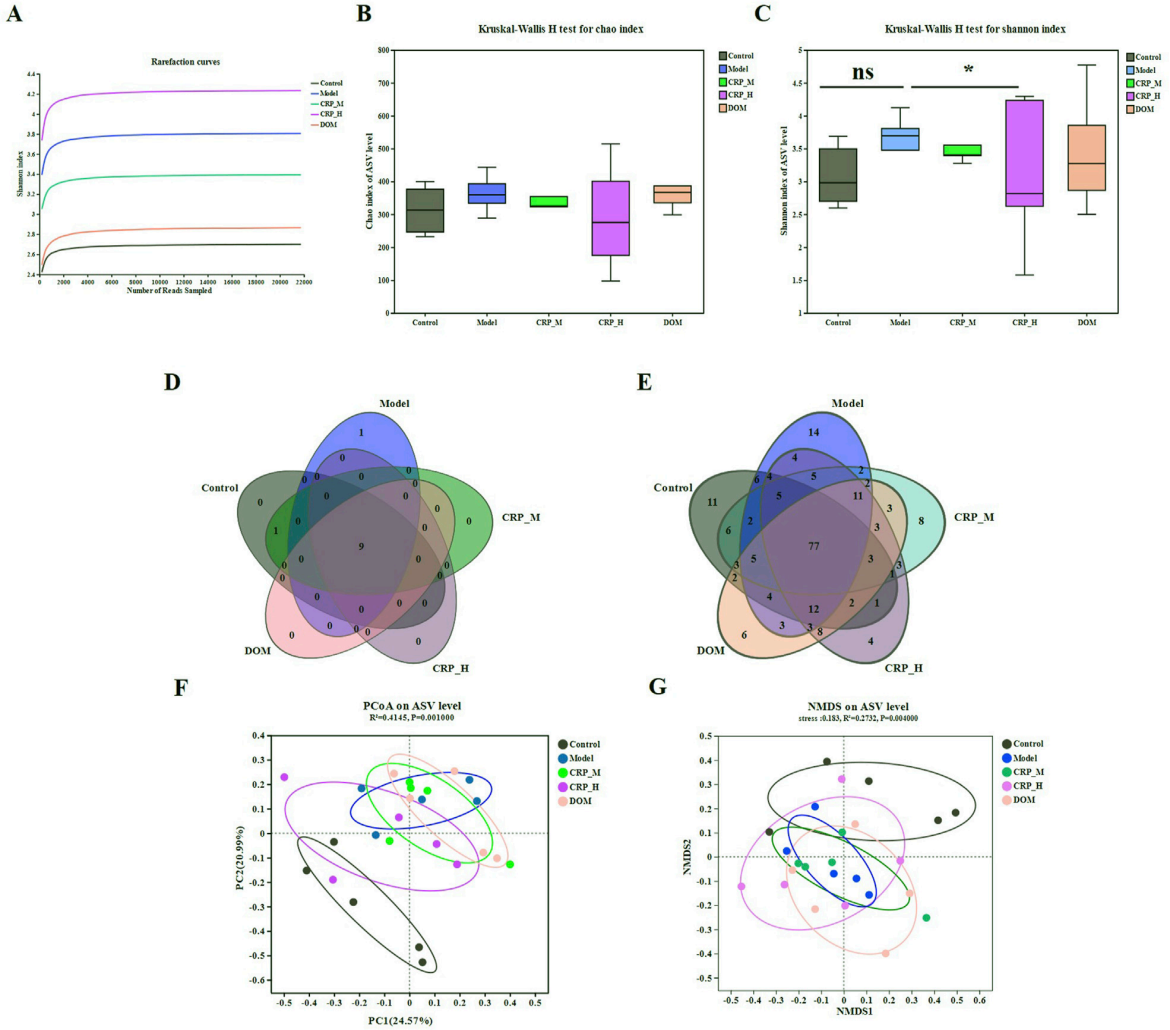
CRP modulated the structure and diversity of the gut microbiota (n = 5). **(A)** Rarefaction curves. **(B)** Chao indices. **(C)** Shannon indices. **(D)** Venn diagram at the phylum level. **(E)** Venn diagram at the genus level. **(F)** PCoA score plots at the species level. **(G)** NMDS score plots at the species level. *P < 0.05 vs. model group.

There is a close relationship between gut microbiota disorder and inflammation. Therefore, we analyzed the composition of the gut microbiota in FD rats. A total of 1,756,138 optimized sequences with an average sequence length of 421 bp were collected after denoising. The number of valid sequences was 21,747 per sample, ensuring that all samples were evaluated at the same sequencing depth after homogenization. As depicted in Figure 7A, the flat intergroup dilution curves indicated that a sufficient amount of sequencing data was available. The Chao and Shannon indices were used to assess the abundance and diversity of the colony community, as well as their increase with higher abundance and diversity (Konopiński, 2020). Compared with the model group, the Shannon value of the CRP-H group significantly increased, while the Chao value showed no significant difference (Figures 7B, C). The results indicated that treatment with CRP could restore both the abundance and diversity of gut microbiota. The species Venn diagram showed that at the phylum level, there are nine common bacteria in the five groups, one unique bacterium in the model group, and one common bacterium in the CRP-M and control groups. At the genus level, there are 77 common bacteria in the five groups, 11 unique bacteria in the control group, 14 unique bacteria in the model group, four unique bacteria in CRP-H, eight unique bacteria in CRP-M, and six unique bacteria in the DOM group (Figures 7D, E). This suggests that CRP could increase the abundance and diversity of the gut microbiota in FD rats. Figures 7F and G show that significant differences in the composition and arrangement of gut microbiota between different groups in β-diversity analysis. These data show a distinct separation between the control, FD, CRP, and positive drug groups. Notably, the CRP-H and control groups displayed the trends and characteristics of mutual proximity in terms of microbiota.

### 3.6 CRP regulates gut microbiota structure and function in FD rats

**FIGURE 8:**
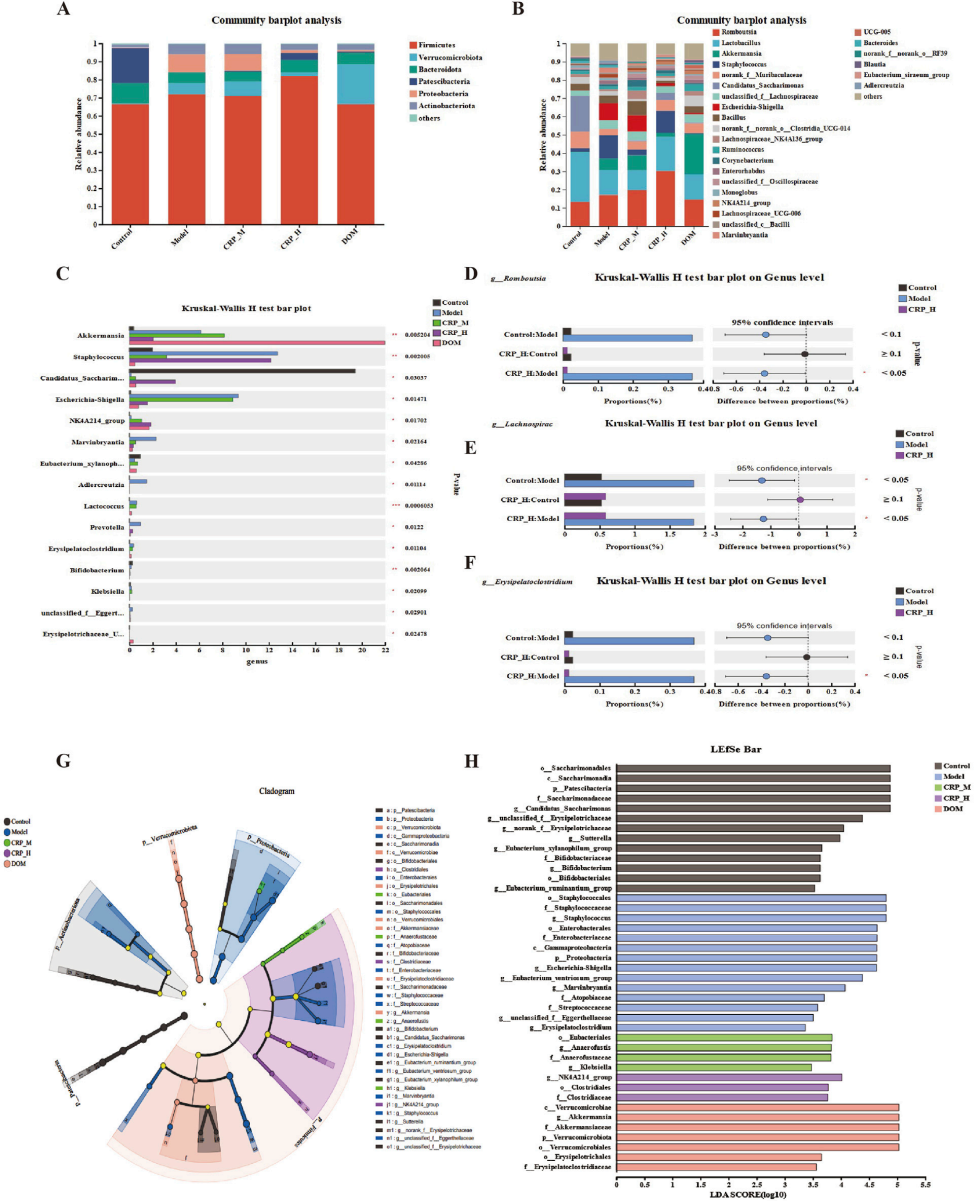
CRP treatment significantly altered the gut microbiota in FD rats. **(A)** Bar plot of community composition at phylum level. **(B)** Bar plot of community composition at genus level. **(C)** Kruskal–Wallis H test bar plot at genus level. **(D–F)** *g__Romboutsia*, *g__Lachnospirac*, and *g__Erysipelatoclostridium* abundance changes. **(G)** LEfSe performed to determine the difference in abundance. **(H)** LDA distribution.

We first estimated the phylum- and genus-level architecture alternation to estimate the role of CRP on gut microbial structure and composition. At the phylum level, we found a significant increase in the abundance of Proteobacteria (9.17%) and Verrucomicrobiota (6.14%) in the model group, while Patescibateria (0.06%), Bacteroidota (1.49%), and Actinobacteriota (0.23%) exhibited a significant decrease compared to the normal group. An increase in Patescibateria (0.35%) and Bacteroidota (2.09%) was observed following CRP treatment, while Verrucomicrobiota (1.09%) and Proteobacteria (0.51%) decreased compared to the model group (Figure 8A). Figure 8B shows a respective proportion at the genus level of a different group. The relative abundance of the top-ten genera included *Romboutsia*, *Lactobacillus*, *Akkermansia*, *Staphylococcus*, *norank_f__Muribaculaceae*, *Candidatus_Saccharimonas*, *unclassified_f__Lachnospiraceae*, *Escherichia/Shigella*, *Bacillus*, and *norank_f__norank_o__Clostridia_UCG-014* as the main probiotics in the intestine, exhibiting better ability to promote the growth and development of intestinal epithelial cells while restoring intestinal homeostasis (O’Callaghan and O’Toole, 2013; Liu et al., 2022). *Lactobacillus* and *norank_f__Muribaculaceae* played an important role in anti-inflammation and was rich in the control rats, while the proportions of *Lactobacillus* (1.27%), *Candidatus_Saccharimonas* (0.08%), and *norank_f__Muribaculaceae* (0.29%) were decreased in FD rats. The FD rats also showed a higher abundance of *Escherichia/Shigella* (5.65%) compared to normal rats, and *Lactobacillus* (4.36%), *candidatus_Saccharimonas* (1.27%), and *norank_f__Muribaculaceae* (2.18%) increased after CRP intervention. *Escherichia/Shigella* (1.04%) decreased compared to the model group. We conducted an intergroup Kruskal–Wallis H test at the genus level, identifying the top 15 abundant and statistically different bacterial groups (Figure 8C). Among these, *g__Erysipelatoclostridium* and *g__Lachnospirac* were significantly increased in the FD rats compared to the normal rats. *g__Romboutsia* abundance was decreased compared to the normal rats, while CRP treatment reversed these situations (Figures 8D–F). The top three biomarkers of gut microbiota in the normal rats included *p_Patescibacteria*, *c_Saccharimonadia*, and *o_Saccharimonadales* (Figures 8G, H). The model group included *g_NK4A214_group*, *o_Clostridiales*, and *f_Clostridiaceae* and the CRP group included *o_Staphylococcales*, *f_Staphylococcaceae*, and *g_Staphylococcus*. In general, FD-induced chronic stress stimulation led to an imbalance in the gut microbiota, whereas treatment with CRP reconstructed the microbiota composition.

## 4 Discussion

FD is a gastrointestinal disorder characterized by the absence of substantial organ lesions, and it adversely affects the quality of life and psychological wellbeing (Enck et al., 2017). In recent years, the widespread prevalence of FD has negatively impacted societal wellbeing. Therefore, there is a necessity for identifying remedies for FD patients that are both more effective and safer. Recent studies have confirmed the distinctive therapeutic benefits of TCM in treating FD (Zhu et al., 2020; Hao et al., 2024). CRP, a well-documented TCM, is recognized for its positive effects on the digestive, respiratory, and cardiovascular systems (Yu et al., 2018). Although there is a large amount of classical literature and reports documenting the beneficial therapeutic effect of CRP on indigestion, its influence and exact mechanism of action are not fully determined.

In our experiment, 90 metabolites were identified in CRP. Among the top-20 metabolites, hesperidin could reverse N-methyl- N-nitro- N-nitroguanidine-induced gastric injury through the PI3K-AKT signaling pathway and cell proliferation (Liang et al., 2022). Nobiletin played an anti-oxidative stress and inflammation role by improving the MAPK signaling pathway (Li et al., 2018). Tangeretin might potentially enhance radiosensitivity and inhibit the radiation-induced epithelial–mesenchymal transition of gastric cancer cells (Zhang et al., 2015). Hirsuteine showed a preventive effect on the development of gastric erosions in mice (Ozaki, 1989). The *Compositae* extraction, rich in cryptochlorogenic acid, highly facilitated the biogenesis of gastric epithelial AGS cells and inhibited the expressions of NF-κB, IL-8, and TNF-α (Marengo et al., 2018). Quercetin could enhance gastric ulcer treatment through the Nrf2/HO-1 and HMGB1/TLR4/NF-κB pathways (Shams and Eissa, 2022), protecting gastric lesions from ethanol (Kahraman et al., 2003). This evidence indicates that CRP extract has a favorable influence on gastric function and anti-inflammation, which is basically consistent with our research results.

Network analysis is a mature approach used to predict potential molecular mechanisms that underlie the interactions between various diseases and TCM, making it easy to identify active metabolites within TCM and serving as a technical tool for the research and development of disease treatment drugs (Chen et al., 2021; Li and Zhang, 2013). In this study, we found 220 targets of the bioactive metabolites in CRP extract and 70 intersection targets as the potential targets in treating FD. Furthermore, we found that the mechanism of CRP in treating FD is likely associated with immune response, nervous system, and inflammation through GO and KEGG enrichment analysis. The PPI network analysis identified MAPK1, HSP90AA1, MMP9, mTOR, ESR1, MAP2K1, PTGS2, EGFR, STAT3, and MAPK3 as potential core targets for CRP extract treatment of FD, which are closely related to inflammation. Therefore, we speculate that CRP’s protective effect against gastric dysfunction may be related to its modulation of inflammatory response through these critical targets. However, further *in vivo* experimental verification is necessary to confirm this speculation.

As we know, weight loss is the typical clinical feature of FD (Tack et al., 2016). In the present experiment, the observed weight loss of rats was consistent with previous literature, indicating that the FD model was successfully established (Zhu et al., 2020). Following treatment, the CRP-M, CRP-H, and DOM could recover the body weight of FD rats. Additionally, reduced inflammation infiltration and no hemorrhage and ulcers were observed in the treatment group. The gastric emptying and propulsive intestinal rates are extremely important processes in promoting food from the stomach into the duodenum, which are also a key link in promoting food absorption (Carbone and Tack, 2014; Kusano et al., 2014). There is some researching indicating that FD patients exhibit gastrointestinal motility disorder, impaired gastric emptying, and slow intestinal motility (Guo et al., 2018; Jeon et al., 2019). In this study, we showed that CRP at 0.4 g/kg could improve the gastric emptying and propulsive intestinal rates.

MAPKs are kinases composed of p38, ERK1/2, and JNK, are primarily responsible for external stress signals and most cellular responses to inflammatory cytokines, and are crucial in regulating various inflammatory mediators (Akanda et al., 2018). Previous studies have demonstrated that CUMS-induced FD is accompanied by upregulated MAPK phosphorylation levels, and that inhibiting the activation of MAPKs is judged to be an ideal molecular target for treating FD (Duan et al., 2022). We found that CRP can reduce the phosphorylation levels of MAPKs, including p38, ERK1/2, and JNK. IL-6 and TNF-α are considered upstream activators and downstream products of the MAPK signaling pathway. Excessive production of IL-6 and TNF-α further promotes the phosphorylation of p38, ERK1/2, and JNK, which play an extremely important role in the pathogenesis of FD (Li et al., 2013). In addition, other studies indicate that the activation of p38 MAPK is associated with gastric epithelial barrier dysfunction (Thakre-Nighot and Blikslager, 2016) and that inactivate p38 MAPK could repair the epithelial barrier function (Oshima et al., 2008). Furthermore, the ERK1/2 pathway is associated with gastric injury and wound healing, and reducing the phosphorylation of ERK1/2 promotes the integrity of gastric mucosal layer cells (Wei et al., 2021). The current findings further confirm that CRP relieves inflammation and epithelial cell loss and promotes gastric mucosal layer cell reconstruction in CUMS-induced FD, which is related to the inactivation of MAPKs.

MAPKs, as the crucial regulatory proteins in the inflammatory cascade, do not act independently. The activated TLR4/MyD88 pathway is recognized as an important promoter of inflammatory response, which is related to the regulated the expression of MAPKs and NF-κB (Puppala et al., 2022). The NF-κB phosphorylation level is regulated by MAPKs, which ultimately affects the release of IL-1β, IL-6 and TNF-α (Yarmohammadi et al., 2021). Our results indicate that inhibiting the TLR4/MyD88 pathway is one of the targets of CRP in treatment with FD. Furthermore, previous studies have confirmed that inflammatory factors can directly or indirectly affect the secretion of brain–gut peptides, such as GLP-1, motilin (MTL), and peptide tyrosine (PYY), which can promote gastric motility (Tu et al., 2020; Kumar et al., 2020). Intestinal permeability and gastrointestinal motility are regulated and controlled by the brain’s secreted peptides, which in turn affect gastric peristalsis and the function of the gut microbiota. The intestinal mucosal barrier is the primary mechanism through which the brain and intestine communicate. In addition, Xue et al. (2017) indicate that repairing the inflammatory infiltration of intestinal mucosal barrier can effectively promote the secretion of brain–gut peptides, and subsequently influence gastric emptying. Therefore, we speculate that CRP may affect brain–gut peptide secretion by improving the inflammatory response of the intestinal mucosal barrier, thereby recovering gastrointestinal motility. The result of MTL, vasoactive intestinal peptide (VIP), and gastrointestinal electrical measurements further support our conclusion (Supplementary Figure 1).

The gut microbiota are considered one of the most relevant mitigating factors affecting the occurrence and development of FD (Wang et al., 2024). Recently, it has been reported that CRP has the ability to regulate the gut microbiota ecology (Zhang et al., 2020). Interestingly, we observed significant changes in the gut microbiota of FD rats, associated with Kim et al. (2024). In the present experiment, the 16S rRNA sequencing results showed that Bacteroidota and Patescibateria were significantly enriched in treatment with CRP group rats. Previous studies have shown that Bacteroidota can inhibit inflammation in dyspepsia models, increase intestinal mucosal barrier function, reduce the activation of NF - κB signals in intestinal epithelial cells, and relieve the symptoms of FD (Hao et al., 2024). Patescibacteria is a probiotic anaerobic bacteria super-phylum found in mammalian feces and intestines, with the ability of biotransformation, regulating host health, and alleviating metabolic syndrome. This study emphasizes the ability of Patescibacteria to alleviate inflammation and metabolic diseases, as well as its unique antibacterial activity against specific microorganisms (Brown et al., 2022). The absence of *Parabacteroides merdae* in the phylum Bacteroidetes has been proven to exacerbate the severity of FD (Labanski et al., 2020). Therefore, we speculate that the anti-inflammatory activity of CRP may be associated with an increase in Bacteroidota and Patescibateria in the intestine.

This study is the first to demonstrate that CRP may attenuate FD through TLR4/MyD88 by regulating the gut microbial structure. Compared with synthetic drugs used to treat FD, CRP, as a drug food homologous product, has fewer side effects and better safety. Furthermore, compared with other TCM therapies, the therapeutic effect of CRP is milder, making it easier for patients to accept. However, there are several limitations. For instance, the detailed mechanisms by which CRP reduces inflammation in FD have not been fully explored. CRP is rich in flavonoids and so cannot be directly absorbed by the intestine and likely digested by gut microbiota. Previous research has indicated that natural flavonoids such as quercetin ameliorate metabolic syndrome by modulating gut microbiota–bile-acid crosstalk in mice (Zhu et al., 2024). Consequently, it is of significant academic interest to investigate whether CRP influences gut microbiota and their metabolites to reduce inflammation response, thereby providing stronger scientific evidence for CRP as a natural product additive to ameliorate FD.

## 5 Conclusion

Our study demonstrates that CRP may help relieve FD symptoms by reducing inflammation. The capacity of FD to mitigate inflammation could stem from its ability to restore intestinal microbiota structure and enhance the presence of microbes that reduce inflammation. These insights could offer a new perspective for addressing the clinical demands associated with drug therapies for FD.

## Data Availability

The data presented in the study are deposited in the NCBI SRA database, accession number SRP562106.
