# Supplementary material for: Network analysis and experimental validation to investigate chenpi against functional dyspepsia through TLR4/MyD88 by regulating the gut microbial structure
Source: Front Pharmacol. 2025 Feb 13;16:1495799. doi: 10.3389/fphar.2025.1495799 (PMC11865038; doi:10.3389/fphar.2025.1495799)
Supplement: Supplementary file 1 [file DataSheet1.pdf]

Supplementary Figure 1

Materials and methods

Enzyme-linked immunosorbent assay

The serum MTL and VIP were detected by ELISA kits according to the manufacturer's instructions. Each sample optical density (OD) values were obtained by a microplate reader, and the concentration of serum MTL and VIP were analyzed by their standard curves, respectively.

Gastrointestinal electric recordings

A BL-420S experimental system biological function analyzer (TaiMeng Technology, Chengdu, China) with a microcomputer including frequency and amplitude of slow wave and spike activity was employed to record the gastrointestinal electrical signal in duodenum and antrum for 1 h.

Result

The result shown that plasma MTL protein level was significantly decreased in the model group compared with the control group, while CRP could reverse this situation **(Supplementary Figure 1A)**. Similarly, the elevated VIP level in serum can also be significantly reversed by CRP **(Supplementary Figure 1B)**. The results are consistent with previous literature reports (Liang et al., 2018). Additionally, we also measured the changes in migrating motor complex (MMC). The results shown that the MMC cycle in the model group disordered and the phase III disappeared in both antrum and duodenum, as shown in **Supplementary Figure 1C**. However, phase III can be re-observed after 14 days of CRP treatment.

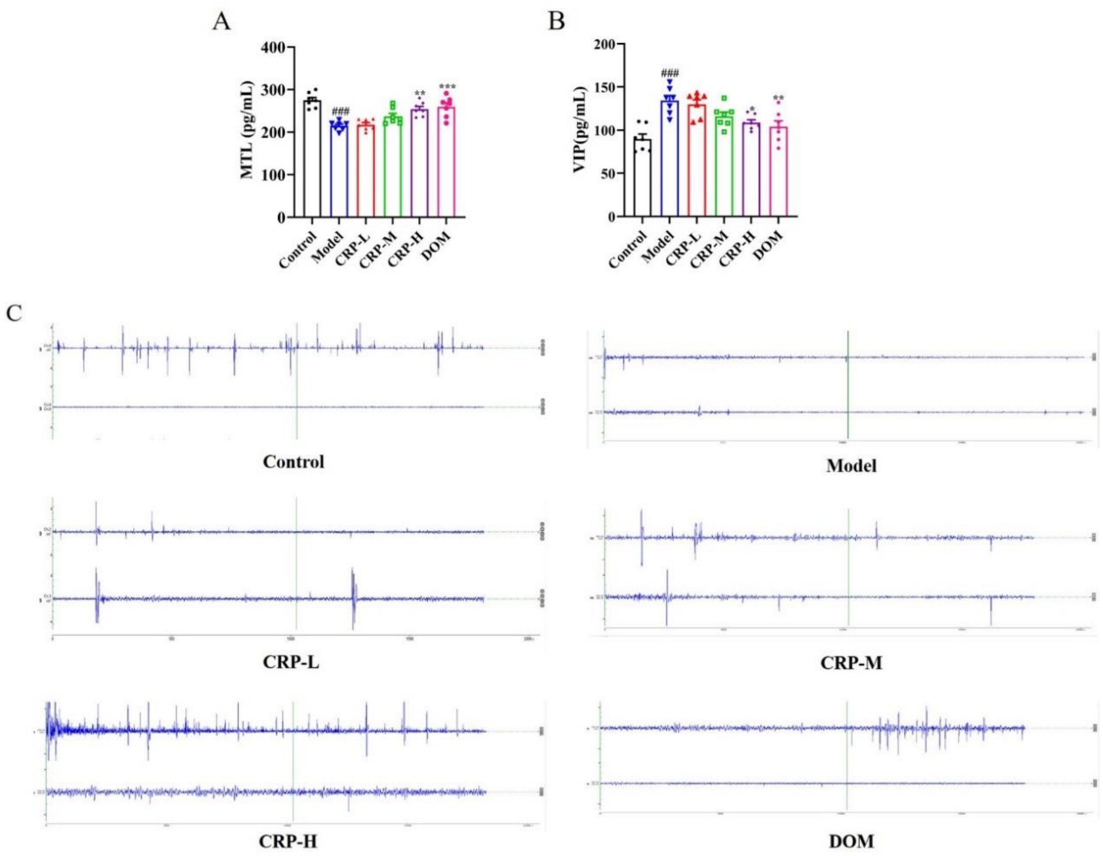

Supplementary Figure 1 Changes in serum brain gut peptide levels and gastrointestinal motility in FD rats. $^{###}p < 0.001$ *vs* Control group; $^{*}p < 0.05$, $^{**}p < 0.01$, $^{***}p < 0.001$ *vs* Model group.
